# Thinking, Feeling, and Moving in Kindergarten Children: How Motor Competence Shapes Executive Function Skills and Emotion Comprehension in Girls

**DOI:** 10.3390/children12101381

**Published:** 2025-10-13

**Authors:** Elena A. Chichinina, Aleksander N. Veraksa, Olga V. Almazova, Linda S. Pagani

**Affiliations:** 1 Faculty of Psychology, Lomonosov Moscow State University, Moscow 125009, Russia; 2 Laboratory of Childhood Psychology and Digital Socialization, Federal Scientific Center of Psychological and Multidisciplinary Research, Moscow 125009, Russia; 3 School of Psycho-Education, University of Montreal, Montreal, QC H3C 3J7, Canada; 4 School Environment Research Group, University of Montreal, Montreal, QC H3C 3J7, Canada; 5 Sainte-Justine’s Hospital Research Center, Montreal, QC H3T 1C5, Canada

**Keywords:** school readiness, executive function skills, motor competence, emotion comprehension, joint activity, kindergarten children

## Abstract

**Highlights:**

**What are the main findings?**

- In girls, high motor competence at 5 years 10 months (Time 1) amplified the relationship between cognitive flexibility at Time 1 and emotion comprehension one year later (Time 2).
- Both verbal and visual working memory at Time 1 predicted emotion comprehension at Time 2; in girls, motor inhibition at Time 1 predicted emotion comprehension at Time 2; and in boys, it was cognitive inhibition.

**What is the implication of the main finding?**

- Joint physical activities, which also challenge executive function skills, may help foster emotion comprehension development.
- The findings highlight the role of motor interventions and joint physical activities when it comes to choosing activities for young children.

**Abstract:**

**Background/Objectives**: Increased screen time partially replaces social interaction, physical activity, and outdoor play in kindergarten children, leading to a risk of decreased cognitive, emotional, and motor skills. Children with high motor skills are more likely to have access to challenging joint activities that promote their cognitive and emotional development. This study examines the moderating role of motor competence in the relationship between executive function skills and emotion comprehension. **Methods**: A sample of 220 kindergarten children (101 girls, 119 boys) completed the NEPSY-II subtests and the ‘Dimensional Change Card Sort’ tool for executive function skills assessment, the Movement Assessment Battery for Children—Second Edition (MABC-2) for motor competence, and the Test of Emotion Comprehension (TEC) for emotion comprehension. Executive function skills and motor competence were assessed when children were in their penultimate year of kindergarten (children were aged on average 5 years 10 months), and emotion comprehension was assessed one year later, when children were in their final year of kindergarten. When children were in their penultimate year of kindergarten, caregivers also reported on children’s passive and active screen time, maternal education, and family income, which were used as control variables. **Results**: For girls, motor competence moderated the relationship between cognitive flexibility and later emotion comprehension. High motor competence amplified this relationship (B = 0.171; SE = 0.066; 95% CI [0.041, 0.302]; *p* = 0.011). For boys, there were no significant moderation effects. **Conclusions**: High motor competence can improve emotion comprehension in kindergarten girls. Emotional development may benefit from effective shared motor interventions for children.

## 1. Introduction

Over the past two decades, sedentary screen time has increased in childhood [1]. As a result, digital play and watching video content have partly replaced social communication, motor activity, and outdoor and dramatic play [2]. Moreover, screen time has also increased in adult caregivers, at the detriment of engaging in stimulating joint activities with children [3]. Reduced quantity and quality of shared adult–child interactions during childhood risk decrements in intellectual, emotional, and motor processing skills in children, which are foundational for healthy personal and academic development in adulthood [4,5].

Executive function (EF) skills represent a group of top-down mental governance processes that regulate one’s thoughts and behaviors [6]. They sustain goal-directed behavior within complex contexts or changing contingencies. EF skills are conceptualized through various models, ranging from unitary to modular frameworks and distinctions like cold and hot EF skills or domain-general and specific EF skills [7,8]. Contemporary theoretical discussions include a skeptical view that the construct of EF skills is overpriced and poorly defined. According to this point of view, EF is an umbrella term for a set of diverse cognitive processes that share a common factor [7]. Consensus in neuroscience, however, highlights three core sub-skills of inhibition, working memory, and cognitive flexibility that are connected by hierarchical relationships [6]. Cognitive flexibility represents the highest level of this hierarchy [9]. It begins to develop actively once inhibition and working memory are established, typically around the age of 4–5 years [6]. During early childhood, EF skills develop rapidly and are crucial for readiness for formal schooling because they regulate healthy growth in cognitive, social, and emotional development [10]. This is particularly important for the foundational development of emotion comprehension skills [11].

Emotion comprehension refers to the ability to process the nature, causes, and consequences of individual emotions and those of others. This understanding helps one manage emotions in daily life. Much like EF skills, emotion comprehension in the early years predicts social and academic performance [12] and personal and professional achievements in adulthood [13]. Early childhood remains a sensitive period for the active development of emotion comprehension [14]. Joint activities with caregivers, through communication and play, represent optimal conditions for emotion comprehension development during early and middle childhood [15].

There is a positive correlation between EF skills and emotion comprehension [16]. This association can be explained by Vygotsky’s cultural–historical [17] and Leontiev’s activity perspectives [15]. According to this approach, EF skills, especially emotion comprehension, develop during shared adult–child activities. The maturation of emotion comprehension is not a spontaneous process, but a purposeful one, and dependent upon experiences of shared cognition with caregivers and play with peers. Alexander Zaporozhets [18], one of the foremost followers of Vygotskian theory, contends that the emergence of new emotions and the ability to understand both personal and others’ emotions are not acquired through passive perception, but through play and learning. Executive functions are essential for participating in shared activities, as they enable children to follow rules and social norms. Among these functions, cognitive flexibility plays a special role because it allows children to overcome cognitive egocentrism, thereby allowing them to engage in rule-based play [19]. Such shared activities are almost impossible without following rules; thus, stronger executive control predicts smoother and more successful participation in shared experiences. Moreover, well-developed EF skills facilitate perspective-taking and emotional intelligence. However, mechanisms that moderate the association between EF skills and emotion comprehension are yet to be fully understood.

Motor competence is often developed during play and learning. It refers to a degree of proficiency in a variety of physical movement skills [20]. In early childhood, it refers to fundamental locomotor, object control, and balance skills [20] in addition to simple manual dexterity skills [21]. This foundational competence forms the basis for the acquisition of more complex movements and sport-specific skills. High motor competence in childhood forecasts physical fitness and movement intensity and frequency [22]. It has also been found to positively predict EF skills [23] and emotional development in children [24]. Nevertheless, the association between motor competence and emotion comprehension, an important indicator of emotional intelligence, warrants investigation at the transition from early to middle childhood.

Optimal motor competence remains essential for socialization and personal development in childhood. Motor competence grows when children learn new foundational movement skills through active leisure and sport. Optimal motor skills, especially manual dexterity skills and fundamental movement skills, predispose children to more opportunities to participate in a variety of shared play experiences (involving object control skills, balance skills, running, jumping) [15]. Accordingly, children who are proficient in fundamental movement skills are likely to have more opportunities to engage in developmentally challenging activities such as sport, play, and games [25]. Children with poor motor competence participate less in play and different physical activities with other children [26]. However, high motor competence alone is insufficient for successful participation in shared activities, which fundamentally rely on EF skills. Thus, optimal participation in developmentally challenging activities with adults and peers requires both advanced motor competence and well-developed EF skills. Based on Vygotsky’s cultural–historical theory [17] and Leontiev’s activity theory, it is plausible that children’s EF skills and emotion comprehension are moderated by motor competence [15]. Therefore, motor competence may strengthen the relationship between EF skills and the development of emotion comprehension.

Through activities related to play and sport, children are exposed to numerous experiences that involve interaction, cooperation, and competition, which allow them to discover and regulate their strengths, emotions, and feelings [26]. In middle childhood, EF skills are positively correlated with motor competence [23], and motor competence is associated with emotion comprehension [27]. However, the moderating role of motor competence in the relationship between EF skills and emotion comprehension has yet to be established [25]. This adds further empirical ground that suggests that motor competence moderates the relationship between EF skills and emotion comprehension in young children.

The literature thus far remains fragmented and is devoted to the relationship between EF skills and fundamental movement skills, the relationship between EF skills and emotion comprehension, or the relationship between fundamental movement skills and emotion comprehension [27]. Motor competence, EF skills, and emotion comprehension have not been sufficiently addressed with an integrated perspective in middle childhood.

One cross-sectional study conducted by Li and colleagues [27] examined the mediating role of EF skills in explaining the relationship between motor competence and emotion comprehension in children aged 3 to 6 years. Both gross motor skills and EF skills were significant predictors of emotional understanding. Remarkably, EF skills mediated the association between gross motor skills and emotional understanding. However, the direct effect of gross motor skills on emotional understanding weakened when EF skills were statistically considered as mediators. Although the findings, based on a limited design, highlight that EF skills, motor competence, and emotion comprehension should be observed as a triadic system, it could be that the association between EF skills and emotion comprehension is moderated by motor competence. Ideally, this must be examined using a longitudinal approach.

Boys and girls experience distinct biological and contextual influences on their cognitive, motor, and emotional development [28]. For example, the content and the structure of the activity of boys and girls are characterized by different behaviors and patterns in social interaction [29]. Among young boys, play tends to involve physical contact and large motor activities (e.g., running, jumping), and is more unstructured yet directed by peers [28]. In contrast, for young girls, play is more structured, verbal, and adult-oriented [29]. Screen time experiences also differ for girls and boys [30]. Differences in content and structure of activities associated with gendered parenting and biological influences may thus lead to differences in the development of EF skills [31], motor competence [32], and emotion comprehension [10] between boys and girls. Research on such variables should ideally adopt a sex-stratified approach, comparing boys with boys and girls with girls.

The purpose of this longitudinal study was to investigate the moderating role of motor competence in the relationship between each EF skill and later emotion comprehension in boys and girls. More specifically, we aimed to examine whether motor skills at age 5 years 10 months moderate the association between EF skills and subsequent emotion comprehension one year later, separately in boys and girls. Potential individual and family characteristics that could influence interpretations, such as screen time, maternal education, and family income, were controlled to provide net estimates above and beyond the influence of competing explanations. Based on respective cultural–historical and activity theoretical perspectives by Vygotsky and Leontiev, we expect that children with higher EF skills will show higher emotion comprehension, and high motor competence will strengthen this link. In addition, we expect that these triadic systems will be different in boys and girls, due to gender differences in the patterns of child development.

## 2. Materials and Methods

### 2.1. Participant Selection and Description

All participants in this IRB-approved study were from Moscow, Russia. The inclusion criteria were as follows: (a) attending kindergarten; (b) typically developing children; and (c) native Russian speakers. Exclusion criteria were as follows: (a) unwillingness to participate; (b) prohibition of sports for health reasons; and (c) absence of sports clothing and footwear for the motor competence assessment. It is important to note that, in Russia, formal schooling (first grade) starts at an average age of 7.5 years, which is one year later than in many countries (e.g., in Europe and North America).

During the first stage of the study (Time 1), in April 2023, 311 children in their penultimate year of kindergarten participated in the assessment of motor competence. At the time of the EF skills assessment (3 and 6 days after motor competence assessment), only 293 of these children were in kindergarten, and 4 children refused to complete the EF skills assessment. At the second stage of the study (Time 2), in March–April 2024, 220 children in their final year of kindergarten with complete motor competence data were available for emotion comprehension assessment. Another 73 children were absent from kindergarten because of illness or shifting to another kindergarten.

This study, therefore, includes 101 girls and 119 boys with complete data on motor competence at Time 1 and emotion comprehension at Time 2. At Time 1, girls were, on average, 5 years and 10 months (±3.7 months) and boys 5 years and 10 months (±3.8 months). At Time 2, girls were, on average, 6 years and 9 months (±3.9 months) and boys 6 years and 9 months (±3.8 months).

### 2.2. Measures

#### 2.2.1. Measures: Predictor Variables—EF Skills (Time 1)

All EF skills, except for cognitive flexibility, were assessed using the NEPSY-II subtests [33]. The NEPSY–II Sentence Repetition subtest measured verbal working memory. Children were required to remember and repeat 17 sentences that progressively increased in complexity, length, and grammatical structure. Two points were awarded for each correctly repeated sentence, one point for a sentence with one or two errors, and zero points for more than two errors (maximum total score = 34). The NEPSY–II Memory for Designs subtest assessed visual working memory. Across four trials that increased in complexity and number of images, children viewed a grid containing pictures and then had to select the appropriate cards and place them on an empty grid in the same locations as previously shown (maximum total score = 120). The NEPSY-II ‘Inhibition’ subtest evaluated cognitive inhibition. Children were asked to name, as quickly as possible, forty figures (squares, circles, and arrows) using the opposite label of what was displayed. Time and error scores were converted into a combined scaled score ranging from 1 to 20. The NEPSY-II ‘Statue’ subtest measured motor inhibition. Children were instructed to remain still for 75 seconds without opening their eyes, speaking, or moving while auditory stimulus were presented. They received zero to two points for successfully following instructions during each 5-second interval (maximum total score = 30). Cognitive flexibility was assessed using the Dimensional Change Card Sort [34]. This task consists of a set of 23 cards featuring blue or red rabbits or boats. In the first trial with six cards, the children sorted the cards by color. In the second trial with six cards, the children sorted the cards by image (rabbits and boats). In the final trial with 14 cards, children alternated between sorting rules based on the presence or absence of a frame on the card (maximum total score = 24).

#### 2.2.2. Measures: Moderator Variable—Motor Competence (Time 1)

Motor competence was assessed using the Movement Assessment Battery for Children-2 (MABC-2) [21]. This included three tasks assessing manual dexterity skills, two tasks assessing object control skills, and three tasks assessing balance. For manual dexterity, children (1) placed plastic coins into a bank box as quickly as possible, (2) threaded beads onto a lace, and (3) traced a single continuous line from point A to point B without crossing the boundaries. For object control, children (4) caught a beanbag thrown by the examiner with both hands and (5) threw a beanbag in an attempt to land it on a designated target mat. For balance, children (6) stood on one leg on a mat for up to 30 seconds with arms free and the opposite foot raised, (7) walked along a line on the floor with heels raised, and (8) made five consecutive jumps from mat to mat, taking off and landing with both feet together. Before each task, the examiner provided a standardized verbal explanation and demonstration, followed by a practice phase with a recommended number of trials. If an error occurred during practice, the examiner reminded the child of the correct procedure. Formal trials commenced immediately afterward, with no assistance provided. The total motor competence score represented the sum of standardized scores across all eight tasks (maximum total score = 149). 

#### 2.2.3. Measures: Outcome Variable—Emotion Comprehension (Time 2)

Emotion comprehension was assessed using the Test of Emotion Comprehension (TEC) [35]. The TEC consists of a picture book, separate versions for girls and boys, which depicts a simple cartoon scenario. Beneath each scenario, there are four emotional outcomes represented by facial expressions. The examiner reads the story aloud, and the child is asked select the emotion that best corresponds to the character’s situation by pointing to one of the four facial expressions. The total score ranges from 0 to 9, with higher scores indicating greater emotion comprehension.

#### 2.2.4. Measures: Confound Control Variables (Time 1)

To account for potential competing explanations, we included parent or caregiver-reported variables, such as family income, maternal education, screen time, and child’s age. Family income was based on the caregiver’s subjective estimate (“below average,” “average,” or “above average”). Maternal education was recorded in total years of schooling. Screen time represented the total number of minutes of passive and active screen exposure per week. Child’s age was measured in months at the time of assessment.

### 2.3. Procedure

At Time 1, motor competence assessment was conducted first, followed by two sessions assessing EF skills three days later. After the assessment, the children’s caregivers completed an online questionnaire about sociodemographic data and the child’s screen time. The questionnaire link was distributed to parents via the kindergarten’s WhatsApp group. At Time 2, only the emotion comprehension assessment was conducted. All the assessments were administered individually with each child and performed by specially trained examiners. During each assessment, the children were allowed to stop the procedure if they did not wish to continue. However, only four children stopped the EF skills assessment, and no one stopped the other assessments. All assessments were carried out between 8 am and 12 pm.

The EF skills assessment was divided into two 20-minute sessions, with a three-day interval between. The first session included tasks assessing verbal working memory and cognitive inhibition. The second session included tasks assessing visual working memory, cognitive flexibility, and motor inhibition. The motor competence assessment was held in the sports hall and took approximately 25–40 min per child. All participants wore their usual sports clothing and footwear during the procedure. The emotion comprehension assessment required approximately 10 min per child. Both EF skills and emotion comprehension assessments were administered in a quiet room familiar to the children within their kindergarten setting. A specially designed application for tablets (Samsung Galaxy Tab A6) was used to administer the EF skills and emotion comprehension tasks. The role of the examiner was only to monitor how each child performed the tasks on the tablet.

The study was approved by the ethics committee of the Faculty of Psychology of Lomonosov Moscow State University (approval No: 2023/18) and conducted in accordance with the Declaration of Helsinki. Caregivers of all participating children were fully informed about the study procedures and provided written informed consent. The MABC-2 was used on a Russian sample for the first time. Therefore, raw scores were converted to standard scores following the procedures outlined in the original MABC–2 Examiner’s Manual [18]. The NEPSY-II subtests, the ‘Dimensional Change Card Sort’ tool, and TEC had been translated and validated for the Russian population [36,37].

### 2.4. Data Analytic Procedures

Our sex-stratified analysis examined whether motor skills moderated the relationship between EF skills and emotion comprehension in kindergarten children. The inferential analyses were computed in Jamovi 2.0.0.0. Descriptive statistics (means, standard deviations, and ranges) were computed for all study variables prior to the main analyses. First, we conducted comparisons between participants having complete and incomplete data. The analysis of the structure of incomplete data revealed that 63.03% of boys and 64.36% of girls had complete data on the moderator and confound control variables. The sex-stratified Levene’s test was not significant for all continuous variables with complete and incomplete data on passive screen time, active screen time, and maternal education. Pearson chi-squared tests were also non-significant for the one categorical variable (family income level) in children with complete and incomplete data, indicating that the data were missing at random. To correct for attrition bias, multiple imputation by chained equations (MICE) was applied in *SPSS* version 23, and five imputed datasets were aggregated for inferential analyses. Second, to examine data structure and rule out multicollinearity prior to moderation analysis, Spearman’s correlations were computed among all study variables. Third, to control for possible third-variable bias, using linear regression, we estimated the relationship between control variables (child and family characteristics) and predictor variables (EF skills) and the moderator variable (motor competence). Finally, moderation analysis tested the hypothetical model in which the association between each EF skill and emotion comprehension depended on the level of motor competence.

## 3. Results

Table 1 reports descriptive statistics for the predictor, moderator, outcome, and control variables in this study before multiple imputation.

Table 2 reports Spearman’s correlations between predictor, moderator, outcome, and control variables separately in boys and girls. It is important to note that correlational analysis of the EF skills measures indicated that cognitive flexibility was the most highly correlated with all other EF skills. Also, correlational analysis revealed no strong associations between motor competence and EF skills, indicating no threat of multicollinearity. The absence of a multicollinearity concern provided the methodological basis for testing a moderation model with EF skills as predictors and motor competence as a moderator variable.

Table 3 reports adjusted unstandardized regression coefficients for the relationship between baseline child and family characteristics (after multiple imputation) and EF skills and motor competence in boys and girls, respectively. In boys, active screen time predicted motor inhibition, maternal education predicted verbal and visual working memory, and the child’s age in months predicted visual working memory, cognitive inhibition, motor inhibition, cognitive flexibility, and motor competence. In girls, active screen time predicted verbal working memory, family income predicted motor competence, and the child’s age in months predicted verbal working memory, visual working memory, cognitive flexibility, and motor competence.

Table 4 reports the relationships between control variables, EF skills, motor skills, and emotion comprehension one year later. In boys and girls, control variables (passive and active screen time, maternal education, family income, child’s age) did not predict later emotion comprehension. For boys, verbal working memory, visual working memory, and cognitive inhibition significantly predicted subsequent increases in emotion comprehension. For girls, verbal working memory, visual working memory, and motor inhibition significantly predicted subsequent increases in emotion comprehension.

We observed one significant interaction, only for girls. Motor competence moderated the relationship between cognitive flexibility at Time 1 and emotion comprehension at Time 2. Higher motor competence amplified the relationship between cognitive flexibility and later emotion comprehension. Table 5 reports the decomposition of the interaction between cognitive flexibility with motor competence levels associated with emotion comprehension in girls.

## 4. Discussion

This study examined the importance of motor competence in kindergarten children. That is, we examined whether motor competence moderates the link between EF skills, at age 5 years and 10 months, and emotion comprehension one year later. Given the sex differences in the development of EF skills, motor competence, and emotion comprehension in early childhood [10,31,32], we employed a sex-stratified approach to our analysis.

We found that among girls, motor competence strengthened the association between cognitive flexibility, measured at Time 1, and subsequent emotion comprehension at Time 2. It might be that girls with high cognitive flexibility and low motor competence may not be involved enough in many joint activities with peers and adults. Therefore, they may not have high emotion comprehension. Such girls are likely to engage in activities that require switching (e.g., academic activities and activities related to needlework and creativity) and therefore have a high level of cognitive flexibility. However, these activities are often sedentary, which do not require motor skills. Also, these activities may not provide a variety of opportunities for shared stimulating activities with others. On the other hand, if a girl has both high motor competence and cognitive flexibility, she has many opportunities to participate in different joint activities. Such girls might be more likely to participate in both verbal and in various motor activities, whereas typically, young girls’ play tends to be more verbal and less physically active compared to boys [28,29]. Thanks to strengths in motor and cognitive flexibility skills, girls might be in a better position to develop emotion comprehension. Cognitive flexibility is necessary for emotion comprehension as the child needs to understand both one’s own point of view and emotions and those of other people at the same time [10]. Moreover, the finding that cognitive flexibility demonstrated the strongest correlations with other EF skills is consistent with prior evidence suggesting its leading role in the development of EF skills at this age. This pivotal role of cognitive flexibility may, in fact, also underlie the observed moderation.

In both boys and girls, verbal working memory and visual working memory at Time 1 predicted emotion comprehension at Time 2. Kindergarten children have already developed abilities that are tested in simple emotion comprehension tasks, such as the ability to recognize and name emotions on the basis of expressive cues, ability to understand how external causes affect the emotions of other people, ability to understand the relation between memory and emotion [14]. Differences between children with low and high emotion comprehension are due to performance in such complex tasks. Visual-imaginative thinking is closely related to visual working memory [38]. And to perform complex emotion comprehension tasks, a child needs to compare the details of the real picture and the image that is formed when listening to the story. Morra and colleagues also found that visual working memory plays an important role in the development of emotion comprehension in children between 5 and 11 years [11]. Li and colleagues revealed that the change of working memory after EF skills training could significantly predict the development of emotional competence in 4-year-old children [16]. Therefore, a child needs to manipulate in working memory multiple aspects of the context and appearance, mimic, pantomimic, and posture of the other person, and then combine this information to comprehend their own emotions and those of others. To understand the emotions of other people, a child needs to listen to this person clearly, so verbal working memory is required. In addition, to understand one’s own emotions, a child needs to be able to name such emotions [39]. Therefore, it is not surprising that verbal working memory and emotion comprehension were related.

Interestingly, girls with higher motor inhibition at Time 1 had better subsequent emotion comprehension at Time 2 compared with girls with lower motor inhibition. Motor inhibition enables children to inhibit irrelevant behaviors, helping them focus on understanding their own emotions and those of others [16]. Conversely, boys with higher cognitive inhibition at Time 1 had better subsequent emotion comprehension at Time 2 compared with boys with lower cognitive inhibition.

These two sex-stratified findings seem natural. Studies indicate that preschool boys naturally tend to be more inclined towards both the amount and intensity of physical activity than girls [40]. Boys’ play is mainly characterized as active and physically assertive, while girls’ play tends to be more verbal and governed by rules set by adults in order to maintain social harmony [28]. The characteristics of boys’ play may foster the development of motor inhibition, while the characteristics of girls’ play may foster the development of cognitive inhibition. It can be assumed that participation in play is inherent not only to one’s own sex, but also to the opposite, and contributes to the development of motor inhibition in girls and cognitive inhibition in boys. Thus, higher motor inhibition in boys and higher cognitive inhibition in girls may be associated with better subsequent emotion comprehension, as these children participate more in play with different characteristics.

Several observations are worthy of note. With the exception of child age, most variables did not significantly predict EF skills and motor competence. We included several confound controls—such as screen time, family income, and maternal education—given the association between child screen time, EF skills, and motor competence [1]. Similarly, studies have shown a link between SES and EF skills and motor competence [41]. Passive screen time was not a predictor of EF skills and motor competence; active screen time was only a predictor of motor inhibition in boys and verbal working memory in girls. A possible explanation is that screen time data was obtained using non-objective measures. SES was also almost not a predictor of EF skills and motor competence: maternal education was a predictor only of verbal and visual working memory in boys; family income was a predictor only of motor competence in girls. The limited predicting role of SES can be explained by the fact that many mothers had higher education and most families had average or above average income level. Moreover, caregivers reported family income level subjectively, meaning that we do not know the real income of the families.

The present study is not without limitations. First, although we found two sex-specific moderator effects that correspond to natural inclinations and gender socialization, we cannot imply causal relationships from our findings due to our associative design. Second, we had no data about adult–child joint activity or other forms of enrichment, as such activities can impact both motor competence and EF skills [42].

In terms of strengths, we used sex-stratified analysis, and this approach respects the idea that boys and girls develop in unique ways because of biological and environmental influences [28]. Furthermore, we controlled for important individual and family characteristics that could influence the interpretation of the findings. Finally, we used tools to assess EF skills, motor competence, and emotion comprehension that are recognized as valid and reliable.

This study offers a better understanding of how motor competence moderates the association between EF skills and emotion comprehension in kindergarten children. The findings can serve as a basis for motor interventions that can improve emotion comprehension. The novelty of our research lies in its longitudinal design, confound control, and our findings that are specific to female development. A better understanding of how these essential elements of early childhood development relate to each other may help educators create effective joint activities for children.

## Figures and Tables

**Table 1:** Descriptive statistics for predictor, moderator, outcome, and control variables.

	Boys				Girls			
	N	Mean	SD	Range	N	Mean	SD	Range
Predictor variable (Time 1)								
Verbal working memory	113	19.2	3.4	12–27	94	19.6	3.4	12–31
Visual working memory	114	71.2	20.5	34–120	94	70.9	19.8	34–120
Cognitive inhibition	117	10.9	3.0	4–18	98	11.1	3.0	5–17
Motor inhibition	115	26.5	3.9	10–30	97	27.2	2.9	14–30
Cognitive flexibility	113	20.1	2.9	10–24	98	20.6	2.8	12–24
Moderator variable (Time 1)								
Motor competence	119	69.9	13.5	30–97	101	73.6	11.8	36–94
Outcome variable (Time 2)								
Emotion comprehension	119	5.9	1.4	2–9	101	6.0	1.3	2–8
Control variables (Time 1)								
Child’s passive screen time, minutes per week	88	536.6	358.6	60–1800	73	549.0	378.4	60–1925
Child’s active screen time, minutes per week	87	163.5	211.1	0–1080	73	171.6	287.1	0–1900
Maternal education	96	16.3	1.4	11–20	81	15.8	1.5	11–20
Child’s age, months	119	70.3	3.8	63–78	101	69.9	3.7	56–79
	N	%			N	%		
Family income	95				82			
below average		5				0		
average		68				78		
above average		27				22		

**Table 2:** Spearman’s correlations between predictor, moderator, outcome, and control variables in boys and girls.

		1	2	3	4	5	6	7	8	9	10	11
2	B	0.230 *										
	G	0.223 *										
3	B	0.184	0.394 ***									
	G	0.149	0.161									
4	B	0.342 ***	0.114	0.015								
	G	0.154	0.075	0.092								
5	B	0.361 ***	0.238 *	0.233 *	0.315 ***							
	G	0.328 **	0.246 *	0.183	0.066							
6	B	0.033	0.100	0.078	0.340 ***	0.133						
	G	0.033	0.364 ***	0.007	0.288 **	0.081						
7	B	0.291 **	0.280 **	0.207 *	0.020	0.128	0.141					
	G	0.284 **	0.260 *	0.171	0.204 *	0.101	−0.049					
8	B	−0.161	0.138	−0.003	−0.188	−0.167	−0.057	−0.153				
	G	−0.102	0.051	0.078	0.030	0.044	0.019	0.157				
9	B	0.002	0.085	0.097	0.017	−0.023	−0.044	−0.051	0.352 ***			
	G	−0.037	0.111	0.021	−0.232 *	0.036	0.027	0.088	0.480 ***			
10	B	0.218 *	0.146	−0.010	0.092	0.075	−0.089	0.088	−0.201 *	−0.182		
	G	0.042	0.046	−0.167	−0.057	−0.059	0.027	−0.142	−0.016	−0.127		
11	B	−0.027	−0.071	0.050	0.188	0.060	0.095	−0.079	−0.006	−0.038	0.140	
	G	−0.024	−0.017	0.103	0.123	−0.209	0.054	−0.014	−0.057	−0.005	−0.081	
12	B	0.047	0.133	−0.216 *	0.172	0.061	0.274 **	0.042	0.045	0.120	−0.246 *	−0.004
	G	0.085	0.097	−0.216 *	0.166	0.234 *	0.123	−0.155	0.126	0.114	0.193	0.020

Notes. B—boys, G—girls; 1—Verbal working memory, 2—Visual working memory, 3—Cognitive inhibition, 4—Motor inhibition, 5—Cognitive flexibility, 6—Motor competence, 7—Emotion comprehension, 8—Passive screen time, 9—Active screen time, 10—Maternal education, 11—Family income, 12—Child’s age; * *p* ≤ 0.05, ** *p* ≤ 0.01, *** *p* ≤ 0.001.

**Table 3:** Unstandardized regression coefficients (standard error) reflecting the adjusted relationship between control variables (screen time, maternal education, family income, child’s age), and EF skills, and motor competence at Time 1 in boys and in girls.

	*B (SE)*					
Control Variables	Predictor Variables					Moderator Variable
	Verbal Working Memory	Visual Working Memory	Cognitive Inhibition	Motor Inhibition	Cognitive Flexibility	Motor Competence
**Boys**						
Passive screen time	−0.001 (0.001)	0.004 (0.003)	0.000 (0.001)	0.001 (0.001)	−0.000 (0.000)	0.000 (0.003)
Active screen time	−0.001 (0.001)	−0.004 (0.004)	−0.001 (0.001)	−0.002 (0.001) *	−0.001 (0.001)	0.001 (0.005)
Maternal education	0.226 (0.107) *	1.803 (0.578) **	0.189 (0.095)	0.178 (0.123)	0.105 (0.079)	−0.031 (0.843)
Family income	−0.405 (0.429)	−1.188 (2.354)	0.439 (0.398)	0.083 (0.473)	0.441 (0.340)	3.159 (2.038)
Child’s age	0.081 (0.042)	1.063 (0.217) **	−0.082 (0.038) *	0.137 (0.046) **	0.063 (0.030) *	0.533 (0.164) **
*R* ^2^	0.053	0.076	0.039	0.052	0.028	0.048
**Girls**						
Passive screen time	0.000 (0.001)	−0.005 (0.003)	0.000 (0.000)	0.000 (0.001)	0.000 (0.000)	−0.001 (0.003)
Active screen time	−0.002 (0.001) *	0.002 (0.004)	0.000 (0.001)	0.000 (0.001)	−0.001 (0.001)	0.004 (0.003)
Maternal education	−0.064 (0.127)	1.159 (0.697)	0.076 (0.098)	0.019 (0.097)	0.106 (0.082)	0.424 (0.977)
Family income	−0.360 (0.446)	2.360 (2.489)	0.672 (0.356)	0.218 (0.387)	0.257 (0.298)	4.238 (1.509) **
Child’s age	0.110 (0.039) *	0.937 (0.210) **	−0.050 (0.033)	0.056 (0.032)	0.067 (0.028) *	0.506 (0.146) **
*R* ^2^	0.038	0.065	0.115	0.010	0.031	0.071

Notes. * *p* ≤ 0.05, ** *p* ≤ 0.01.

**Table 4:** Unstandardized regression coefficients (standard error) reflecting the relationships between each EF skill at Time 1 and emotion comprehension at Time 2, and the moderation effect of motor competence at Time 1 on this relationship in boys and in girls.

	Boys					Girls				
Model with verbal working memory as a predictor										
Model info	*F* = 1.799, *p* = 0.089, *R*^2^ = 0.149					*F* = 1.879, *p* = 0.077, *R*^2^ = 0.176				
	*B (SE)*	*t*	*p*	η^2^p	95% *CI*	*B (SE)*	*t*	*p*	η^2^p	95% *CI*
Verbal working memory	0.108 (0.050)	2.393	0.019 *	0.065	0.018; 0.197	0.120 (0.041)	2.940	0.004 **	0.110	0.039; 0.202
Motor competence	<0.001 (0.011)	0.079	0.938	0.000	−0.021; 0.023	−0.006 (0.012)	−0.524	0.602	0.004	−0.031; 0.018
Verbal working memory * Motor competence	0.003 (0.003)	1.081	0.283	0.014	−0.003; 0.010	−0.004 (0.004)	−1.069	0.289	0.016	−0.012; 0.003
Passive screen time	<0.001 (<0.001)	−1.695	0.094	0.034	−0.002; <0.001	<0.001 (<0.001)	1.484	0.142	0.030	<0.001; 0.002
Active screen time	<0.001 (<0.001)	0.427	0.671	0.002	−0.001; 0.002	<0.001 (<0.001)	−0.466	0.643	0.003	−0.002; <0.001
Maternal education	0.100 (0.119)	0.842	0.402	0.009	−0.137; 0.337	−0.117 (0.100)	−1.173	0.245	0.019	−0.315; 0.082
Family income	−0.454 (0.345)	−1.315	0.192	0.021	−1.141; 0.233	0.199 (0.350)	0.568	0.572	0.007	−0.499; 0.897
Child’s age, Time 1	0.003 (0.003)	1.080	0.283	0.014	−0.003; 0.010	−0.052 (0.047)	−1.113	0.270	0.017	−0.146; 0.041
Model with visual working memory as a predictor										
Model info	*F* = 1.453, *p* = 0.187, *R*^2^ = 0.123					*F* = 1.778, *p* = 0.096, *R*^2^ = 0.169				
	*B (SE)*	*t*	*p*	η^2^p	95% *CI*	*B (SE)*	*t*	*p*	η^2^p	95% *CI*
Visual working memory	0.017 (0.007)	2.328	0.022 *	0.061	0.002; 0.032	0.022 (0.008)	2.921	0.005 **	0.109	0.007; 0.037
Motor competence	<0.001 (0.011)	−0.037	0.970	0.000	−0.023 0.022	−0.016 (0.013)	−1.294	0.200	0.023	−0.042; 0.009
Visual working memory * Motor competence	<0.001 (<0.001)	0.158	0.875	0.000	<−0.001; 0.001	<0.001 (<0.001)	0.692	0.491	0.007	<0.001; 0.002
Passive screen time	<0.001 (<0.001)	−1.967	0.053	0.045	−0.002; 0.000	<0.001 (<0.001)	1.543	0.127	0.033	<0.001; 0.002
Active screen time	<0.001 (<0.001)	0.288	0.774	0.001	−0.002; 0.002	<0.001 (<0.001)	−0.841	0.403	0.010	−0.002; <0.001
Maternal education	0.017 (0.019)	0.142	0.888	0.000	−0.220; 0.254	−0.128 (0.099)	−1.288	0.202	0.023	−0.326; 0.070
Family income	−0.294 (0.350)	−0.841	0.403	0.008	−0.990; 0.401	0.173 (0.352)	0.492	0.625	0.003	−0.529; 0.875
Child’s age, Time 1	−0.004 (0.041)	−0.086	0.931	0.000	−0.086; 0.079	<0.001 (<0.001)	−1.180	0.242	0.020	−0.149; 0.038
Model with cognitive inhibition as a predictor										
Model info	*F* = 1.591, *p* = 0.138, *R*^2^ = 0.129					*F* = 1.106, *p* = 0.369, *R*^2^ = 0.108				
	*B (SE)*	*t*	*p*	η^2^p	95% *CI*	*B (SE)*	*t*	*p*	η^2^p	95% *CI*
Cognitive inhibition	0.135 (0.050)	2.691	0.009 **	0.078	0.035; 0.235	0.086 (0.052)	1.637	0.106	0.035	−0.019; 0.190
Motor competence	−0.002 (0.011)	−0.208	0.835	0.001	−0.025; 0.020	−0.004 (0.013)	−0.291	0.772	0.001	−0.028; 0.021
Cognitive inhibition * Motor competence	0.000 (0.004)	0.278	0.782	0.001	−0.006; 0.008	0.001 (0.005)	0.238	0.813	0.001	−0.008; 0.012
Passive screen time	<0.001 (<0.001)	−1.628	0.107	0.030	−0.002; 0.000	<0.001 (<0.001)	1.104	0.273	0.016	−0.000; 0.002
Active screen time	<0.001 (<0.001)	0.134	0.894	0.000	−0.001; 0.002	<0.001 (<0.001)	−0.012	0.990	0.000	−0.001; 0.001
Maternal education	0.101 (0.117)	0.861	0.391	0.009	−0.132; 0.334	−0.083 (0.107)	−0.777	0.440	0.008	−0.296; 0.130
Family income	−0.438 (0.348)	−1.260	0.211	0.018	−1.130; 0.253	−0.132 (0.370)	−0.358	0.722	0.002	−0.869; 0.605
Child’s age, Time 1	0.040 (0.041)	0.949	0.345	0.010	−0.043; 0.123	−0.061 (0.050)	−1.216	0.228	0.020	−0.161; 0.039
Model with motor inhibition as a predictor										
Model info	*F* = 0.921, *p* = 0.504, *R*^2^ = 0.081					*F* = 1.422, *p* = 0.203, *R*^2^ = 0.138				
	*B (SE)*	*t*	*p*	η^2^p	95% *CI*	*B (SE)*	*t*	*p*	η^2^p	95% *CI*
Motor inhibition	−0.049 (0.043)	−1.124	0.264	0.015	−0.135; 0.037	0.144 (0.061)	2.364	0.021 *	0.073	0.023; 0.266
Motor competence	0.006 (0.013)	0.422	0.674	0.002	−0.021; 0.032	−0.012 (0.014)	−0.770	0.444	0.008	−0.039; 0.017
Motor inhibition * Motor competence	<0.001 (0.003)	−0.064	0.949	0.000	−0.005; 0.004	0.003 (0.004)	0.825	0.412	0.010	−0.005; 0.011
Passive screen time	<0.001 (<0.001)	−1.903	0.060	0.041	−0.002; <0.001	<0.001 (<0.001)	1.020	0.311	0.014	<0.001; 0.002
Active screen time	<0.001 (<0.001)	0.620	0.537	0.005	−0.001; <0.001	<0.001 (<0.001)	0.562	0.576	0.004	0.000; 0.002
Maternal education	0.132 (0.129)	1.025	0.309	0.012	−0.124; 0.389	−0.069 (0.106)	−0.651	0.517	0.006	−0.280; 0.142
Family income	−0.378 (0.368)	1.027	0.307	0.012	−1.111; 0.354	−0.069 (0.373)	−0.185	0.854	0.000	−0.812; 0.674
Child’s age, Time 1	−0.004 (0.043)	−0.103	0.918	0.000	0.089; 0.080	−0.089 (0.049)	−1.828	0.072	0.045	−0.186; 0.008
Model with cognitive flexibility as a predictor										
Model info	*F* = 1.023, *p* = 0.426, *R*^2^ = 0.090					*F* = 2.117, *p* = 0.045, *R*^2^ = 0.188				
	*B (SE)*	*t*	*p*	η^2^p	95% *CI*	*B (SE)*	*t*	*p*	η^2^p	95% *CI*
Cognitive flexibility	0.039 (0.059)	0.665	0.508	0.005	−0.078; 0.157	0.015 (0.055)	0.264	0.792	0.001	−0.096; 0.124
Motor competence	0.003 (0.012)	0.260	0.795	0.001	−0.021; 0.027	0.005 (0.012)	0.418	0.677	0.002	−0.020; 0.030
Cognitive flexibility * Motor competence	0.004 (0.004)	1.009	0.316	0.012	−0.004; 0.012	0.013 (0.004)	3.017	0.004 **	0.111	0.004; 0.021
Passive screen time	<0.001 (<0.001)	−1.914	0.059	0.042	−0.002; <0.001	<0.001 (<0.001)	1.469	0.146	0.029	−<0.001; 0.002
Active screen time	<0.001 (<0.001)	0.658	0.512	0.005	−0.001; 0.002	<0.001 (<0.001)	−0.425	0.672	0.002	−0.002; <0.001
Maternal education	0.102 (0.123)	0.827	0.411	0.008	−0.143; 0.347	−0.084 (0.102)	−0.824	0.413	0.009	−0.287; 0.119
Family income	−0.325 (0.360)	−0.903	0.369	0.010	−1.042; 0.391	−0.205 (0.366)	−0.560	0.577	0.004	−0.934; 0.524
Child’s age, Time 1	0.009 (0.041)	0.226	0.822	0.001	−0.073; 0.091	−0.071 (0.047)	−1.510	0.135	0.030	−0.166; 0.023

Notes. * *p* ≤ 0.05, ** *p* ≤ 0.01.

**Table 5:** Decomposition of the interaction between cognitive flexibility with motor competence Levels at Time 1 associated with emotion comprehension at Time 2 in girls.

Moderator Variable Level	*B (SE)*	*t*	*p*	η^2^p	95% *CI*
Low Motor Competence (Mean − 1 SD)	−0.142 (0.084)	−1.683	0.097	0.037	−0.311; 0.026
Average Motor Competence (Mean)	0.015 (0.055)	0.264	0.792	0.001	−0.095; 0.124
High Motor Competence (Mean + 1 SD)	0.171 (0.066)	2.609	0.011 *	0.085	0.041; 0.302

Notes. * *p* ≤ 0.05.

## Data Availability

The data that support the findings of this study are available from the corresponding author upon request.

## References

[1] Fitzpatrick C., Florit E., Lemieux A., Garon-Carrier G., Mason L. (2025). Associations Between Preschooler Screen Time Trajectories and Executive Function. Acad. Pediatr..

[2] Moore S.A., Faulkner G., Rhodes R.E., Brussoni M., Chulak-Bozzer T., Ferguson L.J., Mitra R., O’Reilly N., Spence J.C., Vanderloo L.M. (2020). Impact of the COVID-19 virus outbreak on movement and play behaviours of Canadian children and youth: A national survey. Int. J. Behav. Nutr. Phys. Act..

[3] Lee S., Kim D., Shin Y. (2024). Screen time among preschoolers: Exploring individual, familial, and environmental factors. Clin. Exp. Pediatr..

[4] Korzeniowski C., Ison M.S., Difabio de Anglat H.A., Gargiulo P.Á., Mesones Arroyo H.L. (2021). Summary of the Developmental Trajectory of Executive Functions from Birth to Adulthood. Psychiatry and Neuroscience Update.

[5] Tervo-Clemmens B., Calabro F.J., Parr A.C., Fedor J., Foran W., Luna B. (2023). A canonical trajectory of executive function maturation from adolescence to adulthood. Nat. Commun..

[6] Diamond A. (2013). Executive Functions. Annu. Rev. Psychol..

[7] Demetriou A., Kazali E., Spanoudis G., Makris NKazi S. (2024). Executive function: Debunking an overprized construct. Dev. Rev..

[8] Sambol S., Suleyman E., Scarfo J., Ball M. (2023). A true reflection of executive functioning or a representation of task-specific variance? Re-evaluating the unity/diversity framework. Acta Psychol..

[9] Souissi S., Chamari K., Bellaj T. (2022). Assessment of executive functions in school-aged children: A narrative review. Front. Psychol..

[10] Mengxia L. (2024). Preschoolers’ cognitive flexibility and emotion understanding: A developmental perspective. Front. Psychol..

[11] Morra S., Parrella I., Camba R. (2011). The role of working memory in the development of emotion comprehension. Br. J. Dev. Psychol..

[12] Richard S., Cavadini T., Dalla-Libera NAngonin S., Alaria L., Lafay A., Berger C., Gentaz E. (2025). The development of specific emotion comprehension components in 1285 preschool children. Sci. Rep..

[13] Belasheva I.V., Ermakov P.N. (2023). Subjective Well-Being of Students with Alexithymia in the Context of Development of their Emotional Competencies during the COVID-19 Pandemic. Natl. Psychol. J..

[14] Pons F., Harris P.L., De Rosnay M. (2004). Emotion comprehension between 3 and 11 years: Developmental periods and hierarchical organization. Eur. J. Dev. Psychol..

[15] Leontiev A.N. (1975). Activity, Consciousness, and Personality.

[16] Li Q., Liu P., Yan N., Feng T. (2020). Executive Function Training Improves Emotional Competence for Preschool Children: The Roles of Inhibition Control and Working Memory. Front. Psychol..

[17] Zinchenko Y.P., Sobkin V.S. (2024). L.S. Vygotsky: History of the future. Lomonosov Psychol. J..

[18] Zaporozhets A.V., Kosheleva A.D. (1985). Nurturing emotions and feelings in preschoolers. Emotional Development of a Preschooler.

[19] Piaget J. (1932). The Moral Judgment of the Child.

[20] Utesch T., Bardid F., Büsch D., Strauss B. (2019). The Relationship Between Motor Competence and Physical Fitness from Early Childhood to Early Adulthood: A Meta-Analysis. Sports Med..

[21] Henderson S.E., Sugden D.A., Barnett A.L. (2007). Movement Assessment Battery for Children-2.

[22] Malambo C., Nová A., Clark C., Musálek M. (2022). Associations between Fundamental Movement Skills, Physical Fitness, Motor Competency, Physical Activity, and Executive Functions in Pre-School Age Children: A Systematic Review. Children.

[23] Willoughby M.T., Hudson K. (2023). Contributions of motor skill development and physical activity to the ontogeny of executive function skills in early childhood. Dev. Rev..

[24] Mohammadi Orangi B., Lenoir M., Yaali R., Ghorbanzadeh B., O’Brien-Smith J., Galle J., De Meester A. (2023). Emotional intelligence and motor competence in children, adolescents, and young adults. Eur. J. Dev. Psychol..

[25] Hill P.J., Mcnarry M.A., Mackintosh K.A., Murray M.A., Pesce C., Valentini N.C., Getchell N., Tomporowski P.D., Robinson L.E., Barnett L.M. (2024). The Influence of Motor Competence on Broader Aspects of Health: A Systematic Review of the Longitudinal Associations Between Motor Competence and Cognitive and Social-Emotional Outcomes. Sports Med..

[26] Martin-Rodriguez A., Gostian-Ropotin L.A., Beltran-Velasco A.I., Belando-Pedreno N., Simon J.A., Lopez-Mora C., Navarro-Jimenez E., Tornero-Aguilera J.F., Clemente-Suarez V.J. (2024). Sporting Mind: The Interplay of Physical Activity and Psychological Health. Sports.

[27] Li Q., Wang Q., Xin Z., Gu H. (2022). The Impact of Gross Motor Skills on the Development of Emotion Understanding in Children Aged 3-6 Years: The Mediation Role of Executive Functions. Int. J. Environ. Res. Public Health.

[28] Berenbaum S., Martin C., Hanish L., Briggs P., Fabes R., Becker J.B., Berkley K.J., Geary N., Hampson E., Herman J.P., Young E. (2007). Sex Differences in Children’s Play. Sex Differences in the Brain: From Genes to Behavior.

[29] Sobkin V., Skobeltsina K. (2015). Shared activities of parents and their preschool children during family pastime. Psychol. Russ. State Art.

[30] Twenge J.M., Farley E. (2021). Not all screen time is created equal: Associations with mental health vary by activity and gender. Soc. Psychiatry Psychiatr. Epidemiol..

[31] Shinohara I., Moriguchi Y. (2020). Are there sex differences in the development of prefrontal function during early childhood?. Dev. Psychobiol..

[32] Smits-Engelsman B., Coetzee D., Valtr L., Verbecque E. (2023). Do Girls Have an Advantage Compared to Boys When Their Motor Skills Are Tested Using the Movement Assessment Battery for Children, 2nd Edition?. Children.

[33] Korkman M., Kirk U., Kemp S. (2007). NEPSY II: Clinical and Interpretive Manual.

[34] Zelazo P.D. (2006). The Dimensional Change Card Sort (DCCS): A method of assessing executive function in children. Nat. Protoc..

[35] Pons F., Harris P. (2000). Test of Emotion Comprehension: TEC.

[36] Veraksa A.N., Almazova O.V., Bukhalenkova D.A. (2020). Executive functions assessment in senior preschool age: A battery of methods. Psychol. J..

[37] Veraksa N.E., Veraksa A.N., Gavrilova M.N., Bukhalenkova D.A., Tarasova K.S. (2021). The Russian Version of the Test of Emotion Comprehension: Adaptation and Validation for Use in Preschool Children. Psychology. J. High. Sch. Econ..

[38] Tong F. (2013). Imagery and visual working memory: One and the same?. Trends Cogn. Sci..

[39] Bukhalenkova D.A., Veraksa ANGuseva U.D., Oshchepkova E.S. (2024). The relationship between vocabulary size and emotion understanding in children aged 5–7 years. Lomonosov Psychol. J..

[40] García J.L., Aadland E., Berger NHansen B.H., Benadjaoud M.A., van Hees V., Danilevicz I.M., Sabia S. (2025). Differences in the activity intensity distribution over the day between boys and girls aged 3 to 17 years. Sci. Rep..

[41] Ming H., Zhang F., Jiang Y., Ren Y., Huang S. (2021). Family socio-economic status and children’s executive function: The moderating effects of parental subjective socio-economic status and children’s subjective social mobility. Br. J. Psychol..

[42] Yang L., Corpeleijn E., Hartman E. (2024). Daily Physical Activity, Sports Participation, and Executive Function in Children. JAMA Netw. Open.

